# Community mental health care in Mexico: a regional perspective from a mid-income country

**DOI:** 10.1186/s13033-020-00429-9

**Published:** 2021-01-11

**Authors:** Jaime Carmona-Huerta, Sol Durand-Arias, Allen Rodriguez, Carmen Guarner-Catalá, David Cardona-Muller, Eduardo Madrigal-de-León, Rubén Alvarado

**Affiliations:** 1 Instituto Jalisciense de Salud Mental, Avenida Zoquipan 1000-A, Zip Code 45170 Zapopan, Jalisco México; 2 Instituto Nacional de Psiquiatría Ramón de la Fuente Muñiz, Mexico City, México; 3 Centro Universitario de Ciencias de la Salud, Universidad de Guadalajara, Sierra Mojada 950 Colonia Independencia, Zip Code 44340 Guadalajara, Jalisco México; 4 Programa de Salud Mental, Escuela de Salud Pública Facultad de Medicina, Universidad de Chile, Santiago de Chile, Chile

**Keywords:** Mental health services, Accessibility, Human resources

## Abstract

**Background:**

Access to mental health care is a worldwide public health challenge. In Mexico, an unacceptably high percentage of the population with mental disorders does not receive the necessary treatment, which is mainly due to the lack of access to mental health care. The community mental health care model was created and has been implemented to improve this situation. In order to properly plan and implement this model a precise situational diagnosis of the mental health care network is required, thus this is a first approach to evaluate the community mental health networks in the state of Jalisco.

**Methods:**

Two components from the EvaRedCom–TMS instrument were used including a general description and accessibility of the community mental health care network. A geographic and economic accessibility evaluation was carried out for the different regions of the state ranging from scattered rural to urban communities using information gathered from health institutions, telephone interviews and computer applications.

**Results:**

Jalisco’s community mental health network includes a total of 31 centers and 0.64 mental health workers for every 10,000 inhabitants > 15 years of age. The mean transportation cost required to access mental health care was 16.25 USD per visit. The time needed to reach the closest mental health center in 7 of the 13 analyzed regions was more than 30 min and the mean time required to reach a prolonged stay center was 172.7 min with transportation cost (taxi, private and public transport) of 22.3 USD. Some marginalized regions in the state have a mean 114 min required to reach the closest mental health care center and 386 min to reach a prolonged stay center.

**Conclusions:**

This first approach to evaluate the mental health networks in Mexico showed that there are multiple barriers to access its care including an unfavorable number of human resources, long distances, and high costs. The identification of Jalisco’s mental health network deficiencies is the first step towards establishing a properly planned community mental health care model within the country.

## Introduction

Access to mental health care is a worldwide public health challenge [1]. In Latin America and the Caribbean, it is estimated that the treatment gap—which refers to the percentage of people who suffer from a disease or disorder and those that do not receive the necessary treatment—is very high. In Mexico, 87.4% of people with a mild mental disorder, 77.9% of those with moderate disorders and 76.2% of those with severe mental disorders, such as schizophrenia or bipolar disorder, do not receive treatment [2]. The deficiencies in access to mental health care in Mexico are fundamentally due to the lack of services and inequity in the distribution of community and outpatient mental health resources within the country [3].

Most people with mental disorders have to overcome several obstacles to access psychological and/or psychiatric care [4]. Some of the main barriers to access mental health care are social stigma and discrimination, comorbidity of mental disorders and non-communicable diseases [5] as well as the presence of additional mental disorders (dual pathology) [6].

Other obstacles are the lack of trained personnel in health centers, but also the lack of financial resources for the transportation to the nearest health center [7]. Regarding the number of specialized human resources, in Mexico, the rate of psychiatrists is 3.71 and 2.23 psychiatric nurses per 100,000 inhabitants [8], while the recommended rate of psychiatrists is 5.0 per 100,000 inhabitants [9, 10]. In addition, the distribution of specialized mental health personnel is uneven throughout the country, with a higher concentration in large cities and very few or almost none in rural areas and marginal states of the country [11]. Other indicators, such as the travel distances required to access a mental health center or the difficulties in obtaining and maintaining pharmacological treatment, are not registered in our country [12]. Regarding the economic barrier, a recent study in South Africa identified that the cost of transfers for the general population to access a psychiatric consultation was 13.3 dollars (USD), without taking into account the cost associated to consultation and prescribed drugs [13].

These barriers in access to health gave rise to the community mental health care model, which has inspired reform processes to ensure mental health care even in the most remote territories, thus improving accessibility to care as a new paradigm [14]. The main objective of the community mental health care model is to promote social reintegration, strengthening outpatient treatments for people with severe mental disorders—such as schizophrenia—and preventing hospitalizations in psychiatric hospitals [15]. In Latin America, the process of transition to community mental health care has been uneven among countries [16]. This model includes the development of outpatient clinics, day hospitals, rehabilitation centers and sheltered homes [17] and has proven to be cost effective since it improves the distribution of health resources, has a greater geographical scope to provide specialized pharmacological treatment and allows the inclusion of psychosocial interventions such as individual and family psychoeducation [7].

For this paradigm to be properly implemented, a precise situational diagnosis is required to allow the development of improved strategies in public mental health, recognizing the obstacles and deficiencies in the quality of care received by patients [17]. Therefore, the need for tools to evaluate and establish a situational diagnosis in mental health services becomes essential. There are different instruments for the evaluation of community mental health networks, such as the Description and Evaluation of Services and Directories in Europe for Long-Term Care (DESDE-LTC) [18] and the instrument EvaRedCom–TMS [17]. Since the EvaRedCom–TMS instrument is faster to apply and assesses the accessibility (geographic and economic) to existing mental health services [19], we decided to use this instrument to describe the community mental health networks in the state of Jalisco, Mexico.

To our knowledge, this is the first approach to visualize community mental health networks in the state of Jalisco, taking relevance by providing information to establish public mental health strategies to improve access and mental health services through the adequate allocation of human resources and planning for the location of community mental health centers [15].

## Material and methods

### Description of the state and its health regions

**Fig. 1:**
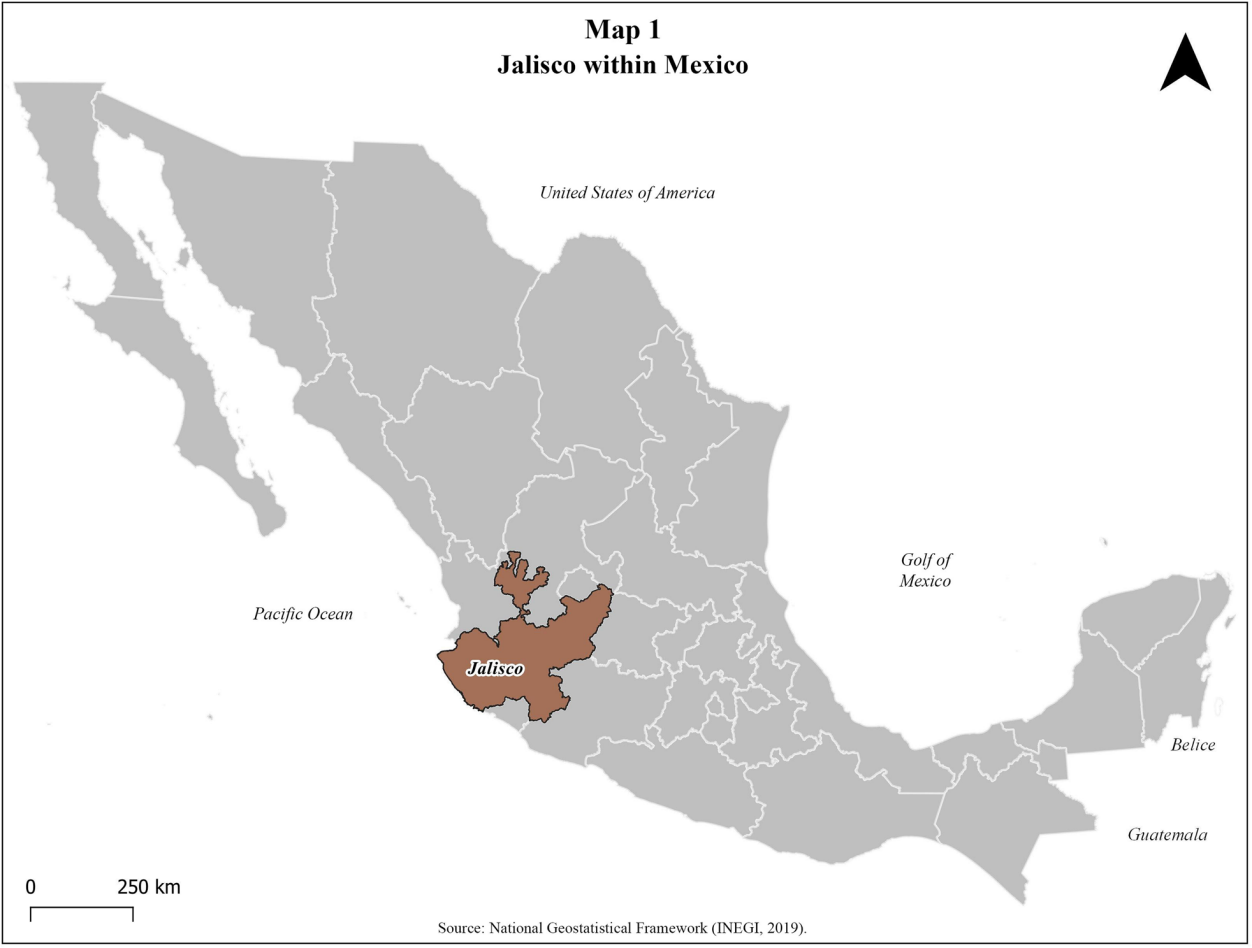
Jalisco within Mexico

Mexico is divided into five mesoregions made up of several federative entities. The State of Jalisco is located in the central-western region with a territorial extension of 78,599km^2^ (Fig. 1). It contains the second largest Metropolitan area in the country: The Metropolitan Area of Guadalajara (ZMG, for its acronym in Spanish).

**Fig. 2:**
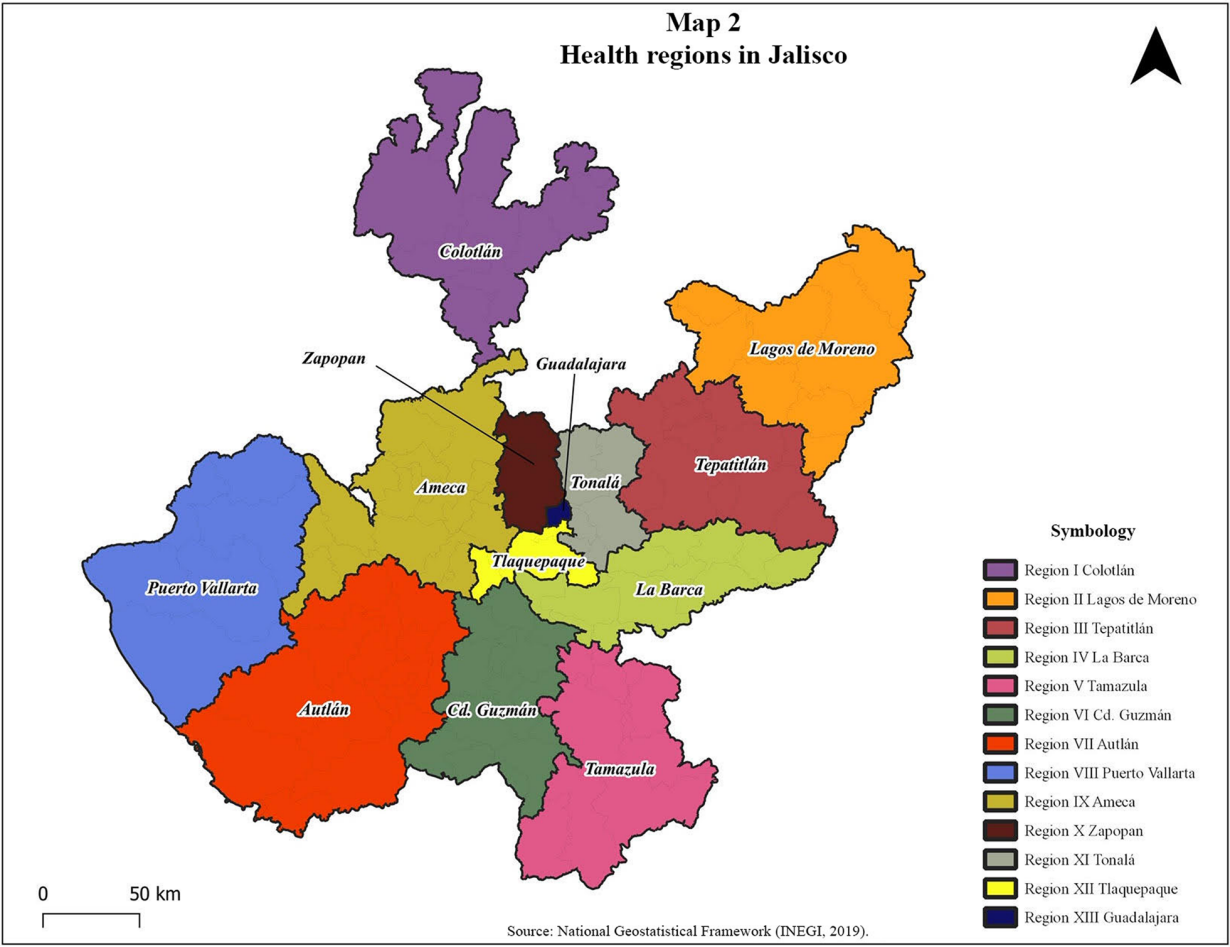
Health regions in Jalisco

Jalisco is made up of 125 municipalities, which in turn comprise 13 geographic and health regions (Fig. 2). Each health region has a main municipality or city and several municipalities, as shown in Table 1.

**Table 1:** Description of the State of Jalisco

Region	Location in Jalisco	Population > 15 years	Municipalities	Type of municipality
1: Colotlán	North	62,321	10	Rural Semi-urban
2: Lagos de Moreno	Altos Norte Region	305,538	12	Rural Semi-urban Intermediate urban Metropolitan urban
3: Tepatitlán	Altos Sur Region	308,655	13	Rural Semi-urban Intermediate urban Metropolitan urban
4: La Barca	Ciénega Region	400,370	13	Rural Semi-urban Intermediate urban
5: Tamazula	Southeast	93,515	10	Scattered rural Rural Semi-urban Intermediate urban
6: Ciudad Guzmán	South	268,731	16	Rural Semi-urban Intermediate urban
7: Autlán	South Coast	231,182	19	Scattered rural Rural Semi-urban Intermediate urban
8: Puerto Vallarta	North Coast	283,684	6	Rural Semi-urban Intermediate urban Metropolitan urban
9: Ameca	Valles Region	293,009	17	Rural Semi-urban Intermediate urban
10: Zapopan	Center	1,039,809	2	Rural Semi-urban Metropolitan urban
11: Tonalá	Center	618,232	6	Rural Semi-urban Intermediate urban Metropolitan urban
12: Tlaquepaque	Center	982,681	5	Rural Semi-urban Intermediate urban Metropolitan urban
13: Guadalajara	Center	1,169,538	1	Metropolitan urban

Scattered rural: less than 2500 inhabitants

Semi-urban rural: 2500 to 15,000 inhabitants

Intermediate urban: 15,000 to 100,000 inhabitants

Urban: more than 100,000 inhabitants [20]

The state has a population of 8,256,000 inhabitants, of which 6,057,265 are people over 15 years of age. Because the state's mental health system is aimed at the population over 15 years old, this study considers this population as the object of study.

### Mental health care in Mexico: conceptual definitions

Mental health services in Mexico are divided into different levels of care. The closest to community mental health care corresponds to the Comprehensive Mental Health Centers (CISAMEs), the second level to outpatient mental health care services in community and general hospitals, and the third level to psychiatric hospitals where outpatient care, and in some cases inpatient care, is provided. In Jalisco, the latter are the Center for Comprehensive Care in Mental Health for Short Stay (CAISAME-EB) and the Center for Comprehensive Care in Mental Health for Long Stay (CAISAME-EP).

The state of Jalisco has eight of the 54 Comprehensive Mental Health Centers (CISAMEs) in the country, being the state with most centers; the two states ranking second have four centers each. It is worth mentioning that eight states of the country do not have any CISAME, even in states with a greater geographic extension than Jalisco.

### Instrument description

There are different instruments for the evaluation of community mental health networks, such as the Description and Evaluation of Services and Directories in Europe for Long-Term Care (DESDE-LTC) designed for the description and evaluation of health services for people with disabilities, being currently one of the most complete models [18]. A second instrument is the EvaRedCom–TMS, which was created in Chile to make a rapid evaluation of community mental health services in low and mid-income countries [17]. Its application is based on data that is easy to collect, such as access to care with distances, times and costs, along with information regarding resources and other specific indicators that are useful for the Assessment Instrument for Mental Health Systems (WHO- AIMS). For this reason and the fact that it has been used and validated in low and mid-income countries like Mexico, we decided to use this tool.

For this study, we have included 2 of the 4 components from the EvaRedCom instrument which include the following:

- A general description of the community care network including the institutions that make up the network, the human resources of each institution and the number of hours available to provide care considering the general population to which these services are available.
- The accessibility to the community care network which includes information on geographic accessibility, that is, travel times, expressed in minutes and hours, to mental health services and the transportation cost of the round-trip to the mental health care center, expressed in dollars (19.90$, value as of December 2019).
- The third component which includes the prevalence of people with Severe Mental Disorders within the community care network was not included due to the fact that there is a void regarding the information required to complete this section especially considering the nonurban prevalence of mental illness.
- The fourth component contains the information regarding the coordination and operation of the community network, including periodic reviews, technical advice of the network, its management, activities that are developed and a rating of the network coordination strategy [17].

### Source of information

The general description of the community care network was obtained through the health centers databases, which depends on the State Health Secretariat. The main health center of each municipality was taken as a reference and starting point for the analysis of geographic and economic accessibility.

Through direct contact with health institutions, information was requested regarding the number of human resources in mental health (a rate was calculated per 10,000 inhabitants with > 15 years of age) and the hours available for clinical care per week (7.5 h/day and 37.5 h/week for each professional).

### Geographic accessibility and transportation costs

The geographic and economic accessibility evaluation was carried out under three methodologies: A (from region one to nine), B (region 10 and 13) and C (region 11 and 12), which are explained below:

- Methodology A: for the health regions are located within the state. These are characterized by having a main municipality that is usually the largest and several nearby municipalities. In these regions there is great mobility in the search for resources, services (including health services) or goods, representing a significant amount of time and economic resources spent. These regions have scattered rural, semi-urban rural and intermediate urban municipalities, and may even have urban municipalities.
- Methodology B: these are the main urban cities found within the ZMG, so mobility is urban, with multiple public transport services and various private options. These concentrate the largest amount of services, goods and resources. It is made up of populations greater than 100,000 inhabitants.
- Methodology C: these two regions have large cities within the ZMG and rural municipalities outside of this area, so it was decided to use both methodologies (A and B).

#### Geographic accessibility

This section refers to the time it takes a user to get to the nearest mental health service center, or one with a higher level of care, from anywhere in the state, both by public transport (bus) and private transport (car). The information was collected through telephone interviews, data provided by health institutions and computer tools such as Google Maps, Waze and Rome2rio. The route from one point to another (from a health center to a mental health service) was introduced between 8 and 13hrs, obtaining an arithmetic mean of the three computer tools, the result is expressed in minutes.

#### Transportation costs

This section considers the traveling cost required to get from anywhere in the state to the nearest mental health service center, or to one with a higher level of care. For this section, all the mobility options that the population may have were taken into consideration, taking into account the costs of public transport (bus), private transport (taxi, Uber) and private transport (car). The information on the costs of public bus transport was collected through the experience of the workers on the different health institutions and telephone calls to bus terminals, with the information being corroborated with prices found on the internet. The information on private transport (taxi, Uber) was made through the costs referred by the taxi terminals and through the Uber mobile application at different schedules. The costs of private transportation (car) were calculated by estimating gasoline consumption per kilometer of an average car, multiplied by the kilometers traveled on a round trip. For this calculation we considered the cost of a liter of regular gasoline to be1 USD, which yields 15 km/l.

## Results

The community mental health network in Jalisco has a total of 31 centers with services for mental health care. It is made up of 13 modules of mental health care in charge of the Ministry of Health, 8 Comprehensive Mental Health Centers (CISAMEs), 5 community hospitals, 3 general hospitals and 2 psychiatric hospitals (Center for Comprehensive Attention in Mental Health of Short Stay (CAISAME-EB) and the Center for Comprehensive Care in Long-Stay Mental Health (CAISAME-EP).

Of the 125 municipalities in the state, 23 have at least one mental health care center (18.4%). This network is made up of 458 mental health workers (Table 2), who serve a total of 6,057,265 inhabitants over 15 years of age and work a total of 17,171 h per week. Therefore, there is one mental health worker for every 13,225 inhabitants over 15 years of age, or in other words, the rate was 0.64 mental health workers for every 10 thousand inhabitants over 15 years of age. There is 1 h of mental health care a week for every 353 inhabitants. On average, there are 137 h of mental health care a week for each of the 125 municipalities and 830 h a week for each of the 13 regions of the state.

**Table 2:** Human resources and hours of mental health care

Region	Population over 15 years	Mental Health care workers	Rate of mental health workers per 10,000 population > 15 years	Hours/week
1: Colotlán	62,321	10	1.60	376
2: Lagos de Moreno	305,538	4	0.13	142
3: Tepatitlán	308,655	9	0.29	340
4: La Barca	400,370	14	0.35	531
5: Tamazula	93,515	4	0.43	152
6: Ciudad Guzmán	268,731	12	0.45	452
7: Autlán	231,182	21	0.91	793
8: Puerto Vallarta	283,684	7	0.25	264
9: Ameca	293,009	8	0.27	301
10: Zapopan	1,039,809	151	1.45	5651
11: Tonalá	618,232	9	0.15	339
12: Tlaquepaque	982,681	181	1.84	6,777
13: Guadalajara	1,169,538	28	0.24	1053
Total	6,057,265	458	0.64	17,171

**Fig. 3:**
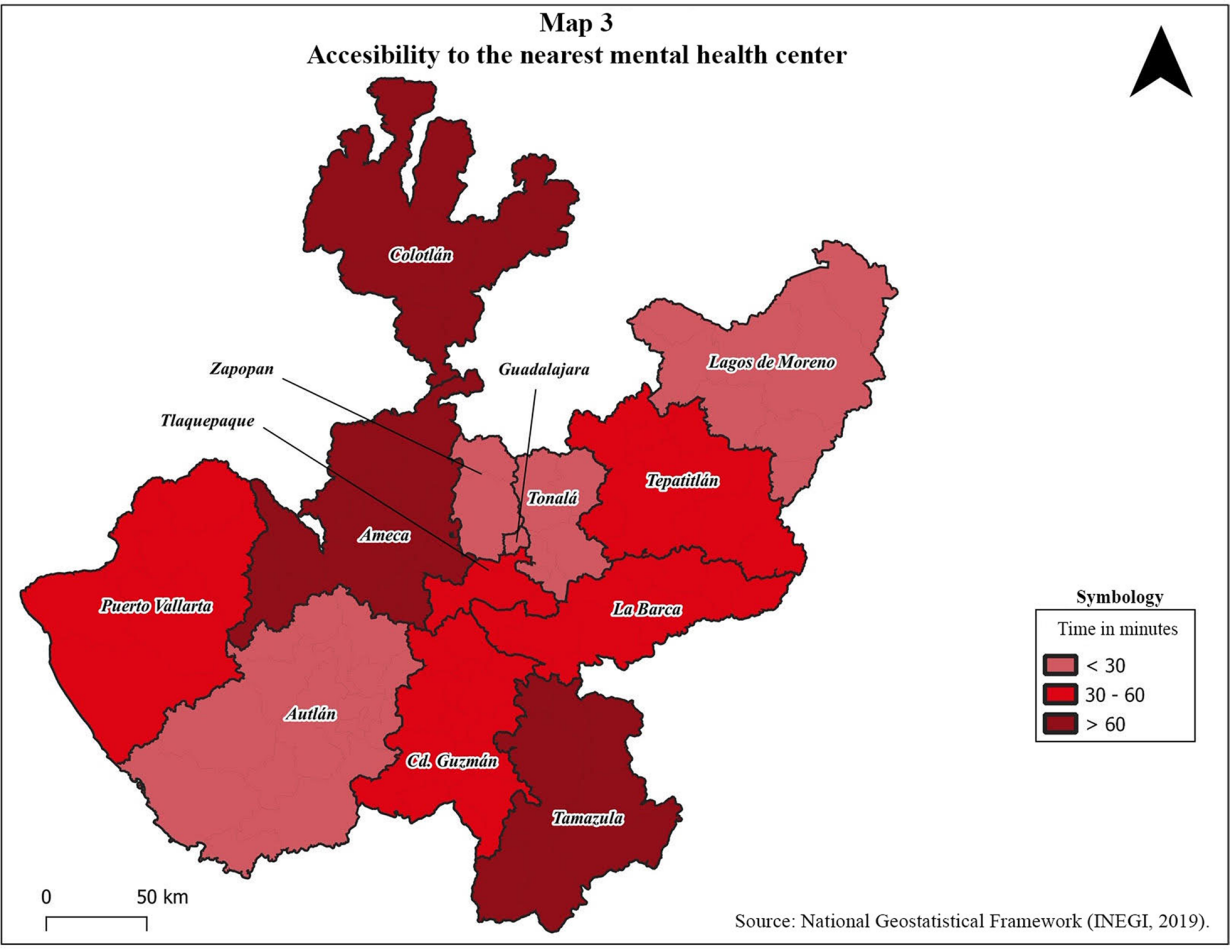
Accessibility to the nearest mental health center

**Fig. 4:**
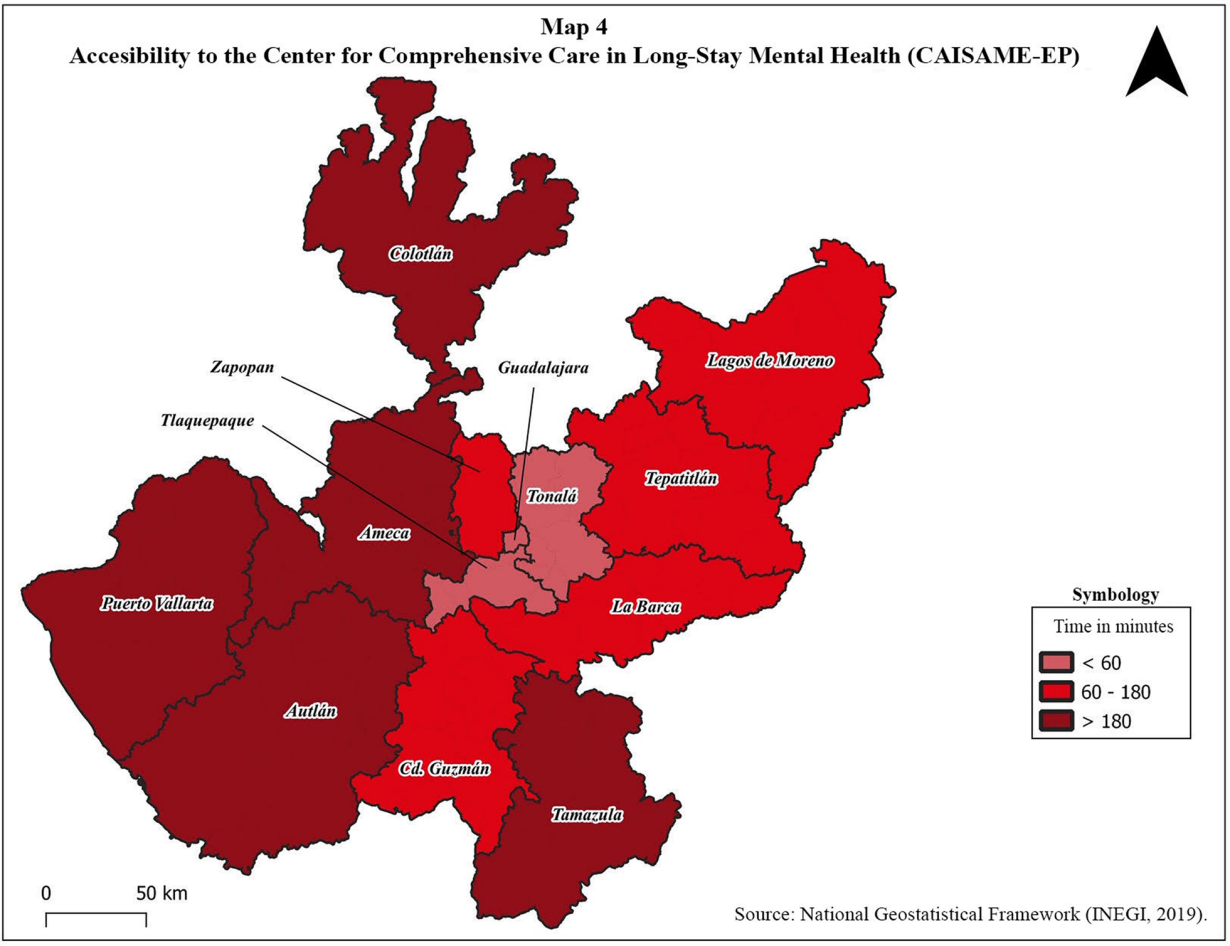
Accessibility to the center for comprehensive care in long-stay mental health (CAISAME-EP)

Regarding time in minutes and costs in dollars that are required to travel, whether by public or private transport, from the head of municipality to the nearest mental health institution and to the CAISAME-EB hospital and the CAISAME-EP hospital, the following weighted means were obtained (Table 3, Figs. 3, 4).

**Table 3:** Times and costs of transfers to mental health institutions

	Nearest mental health center		CAISAME-EB		CAISAME-EP	
Region	Duration (minutes)	Cost (dollars)	Duration (minutes)	Cost (dollars)	Duration (minutes)	Cost (dollars)
1: Colotlán	114	9.5	342	39.2	386	42.6
2: Lagos de Moreno	26	4.8	167	33.1	161	32.2
3: Tepatitlán	32	6.4	141	26.5	114	26.9
4: La Barca	30	4.7	134	25,3	143	22.8
5: Tamazula	68	9.2	216	30.6	223	28.2
6: Ciudad Guzmán	42	7.1	161	23.4	152	25.6
7: Autlán	27	5.9	314	35	280	34.8
8: Puerto Vallarta	42	5.2	369	44.6	356	48.2
9: Ameca	78	6.6	244	15.3	234	6.6
10: Zapopan	26	3	36	3.1	63	7.5
11: Tonalá	29	3.4	57	6.2	47	6
12: Tlaquepaque	35	2.5	64	4.3	37	4.2
13: Guadalajara	21	2.7	36	2.9	50	5.2

	Mean time	Mean cost	Mean time	Mean cost	Mean time	Mean cost
	43.8	5.4	175.4	20.8	172.7	22.3

Table 3 shows a wide variability in the access times to the different mental health centers, as well as in transportation costs. The closest mental health care centers show a mean for geographic accessibility of 43.8 min, with only 5 of the 13 regions having a time of less than 30 min (Fig. 3). Regarding costs, there is an average cost of 5.4 USD, with prices going as low as 2.5 USD and as high as 9.5 USD.

Regarding access to a psychiatric hospital (CAISAME-EP), when hospitalization is needed, Table 3 and Fig. 4 show a reduced geographic accessibility, since only four regions have a time travel less than 60 min (regions 10, 11, 12 and 13). Regarding costs, Table 3 shows a mean cost of 22.3 USD with the lowest cost being 4.2 and the highest being 48.2 USD.

## Discussion

This study is a first approach to the analysis of mental health human resources, geographic accessibility and transportation costs to mental health care in Mexico. The deficiencies described in the state of Jalisco are extrapolated, multiplied and aggravated in other large areas of the country where there are no comprehensive mental health care centers, leaving a large part of the country's population without access to mental health care [21] thus, explaining an important part of the treatment gap.

The global rate of mental health workforce is 9.0/100,000 inhabitants (including all psychologists, psychiatrists, psychiatric nurses and social workers). In the American continent, this same rate is estimated at 10.9/100,000 inhabitants [10]. The results obtained in this study show that the rate of mental health workers in Jalisco ranges from 0.13/10,000 inhabitants > 15 years in the Lagos de Moreno region, to 1.84/10,000 inhabitants > 15 years in the region from Tlaquepaque. Showing that the rate of the mental health workforce in Jalisco is well below the global rate.

Our results regarding time in minutes it takes to get from a community to the nearest mental health center by car or bus reflect that 7 of the 13 regions analyzed required more than 30 min, with an average of 43.8 min. The average time to access one of the two CAISAMEs was very high, with an average of almost three hours by car or bus. The world standards of response time in psychiatric emergencies stipulate that it should not exceed 10 min of waiting, for "semi urgent" cases (potentially dangerous emotional crisis) one hour of waiting and for "non-urgent" cases (follow-up consultation, symptoms exacerbated without risk) up to 2 h of waiting [22]. The fact that the average access time to the nearest mental health center exceeds 30 min reflects that access to mental health is well beyond what is expected, generating an extra risk for psychiatric emergencies, but also complicating care and monitoring of non-urgent cases.

The arithmetic mean of transportation costs to access mental health care (this includes access to the nearest mental health care center, CAISAME-EB and CAISAME-EP) was 16.1 dollars, with a range of 2.5 to 48.2 dollars, being a higher average than that reported in the South African study. With respect to this indicator, there is no data at the global or local level of what the average ideal cost should be. It is considered that the costs to obtain a service or health care should not represent a sacrifice for the personal or family finances, nor be a barrier to access to mental health care.

We emphasize the specific case of Colotlán (the most marginalized area of the state and one of the most marginalized in the country). Although it is one of the regions with the best rates of mental health workers in the state, its location and difficult access means that both times (114 min) and costs (9.5 USD) are extremely high, even for the nearest mental health care center. In the event that hospital care is required, access to CAISAME-EP involves a total of 386 min and an expense of 42.6 dollars. The elements described here highlight the importance of generating, in regions like these, public policies that bring mental health care to marginalized areas, with telepsychiatry being a viable option.

This example reflects the reality of Mexico, in which the distribution of human resources and mental health care are centralized in the main cities and a large population is left at a disadvantage. It should be noted that, in our study, only mental health workers who work in the public sector were counted, excluding those in the private sector, which is why there is such a marked difference in the rates reported in this study compared with what was published by Heinze et al [8].

It is important to highlight that obtaining the information provided here was a challenge. The search for the appropriate methodology due to the demographic, geographical and cultural differences of the various municipalities of the state and limited cooperation coupled with long bureaucratic processes for obtaining information made this a monumental task. The limited previous research carried out in the region, the deficiency in the statistical records, and the segmentation of the Mexican health system were also important barriers to overcome while carrying out the study.

As previously noted, EvaRedCom is an instrument designed to have a rapid evaluation of community mental health services, which is why the main limitation of this work is the precision of the estimates, especially those of geographic accessibility. Another limitation lies in having considered only the most conventional means of transport by the studied population, therefore, other means of transport are not considered and these and special populations with different capacities that require greater support for their transportation, that present greater difficulties and expenses in seeking access to health services are not represented in the present study. For the calculation of the route a person takes to reach specialized mental health services, the main health center in each municipality was used as a starting point. Therefore, there is an underestimation for all the rural and more remote areas, in which people must make previous trips to get to these centers, generating longer times and additional transportation costs. However, previous studies [17] have found that this approximation reflects good variability and that the most remote areas (with greater problems of geographic and economic accessibility) exemplify the contrast with urban areas, thus, fulfilling the function to provide needed information to make good, informed decisions regarding community service allocation.

## Conclusions

The results obtained show that, despite the fact that Jalisco is one of the states with the largest community mental health infrastructure in Mexico, there are still multiple barriers to access its care, identified by the unfavorable number of human resources, distances and costs. This first approach to the evaluation of mental health networks in Mexico allows us to recognize the current situation and consider the factors that must be taken into account for the extension of the community mental health care model in the country, through evidence-based management that allows improving the access to mental health services through proper allocation of human resources and planning for the location of community mental health centers.

## Data Availability

The datasets used and/or analyzed during the current study are available from the corresponding author on reasonable request.

## Abbreviations

DESDE-LTC: Description and Evaluation of Services and Directories in Europe for Long-Term Care
WHO-AIMS: Assessment Instrument for Mental Health Systems
ZMG: Metropolitan Area of Guadalajara
CAISAME-EB: Center for Comprehensive Care in Mental Health for Short Stay
CAISAME-EP: Center for Comprehensive Care in Mental Health for Long Stay
CISAMEs: Comprehensive Mental Health Centers
